# The Effect of Global Spread, Epidemiology, and Control Strategies on the Evolution of the GI-19 Lineage of Infectious Bronchitis Virus

**DOI:** 10.3390/v16030481

**Published:** 2024-03-20

**Authors:** Giovanni Franzo, Giulia Faustini, Claudia Maria Tucciarone, Francesca Poletto, Francesca Tonellato, Mattia Cecchinato, Matteo Legnardi

**Affiliations:** Department of Animal Medicine, Production and Health (MAPS), University of Padua, Viale dell’Università 16, 35020 Legnaro, Italy; giulia.faustini.1@phd.unipd.it (G.F.); claudiamaria.tucciarone@unipd.it (C.M.T.); francesca.poletto.1@studenti.unipd.it (F.P.); francescaromana.tonellato@studenti.unipd.it (F.T.); mattia.cecchinato@unipd.it (M.C.); matteo.legnardi@unipd.it (M.L.)

**Keywords:** IBV, evolution, phylodynamic, environments, natural selection, vaccine, immunity

## Abstract

The GI-19 lineage of infectious bronchitis virus (IBV) has emerged as one of the most impactful, particularly in the “Old World”. Originating in China several decades ago, it has consistently spread and evolved, often forming independent clades in various areas and countries, each with distinct production systems and control strategies. This study leverages this scenario to explore how different environments may influence virus evolution. Through the analysis of the complete S1 sequence, four datasets were identified, comprising strains of monophyletic clades circulating in different continents or countries (e.g., Asia vs. Europe and China vs. Thailand), indicative of single introduction events and independent evolution. The population dynamics and evolutionary rate variation over time, as well as the presence and intensity of selective pressures, were estimated and compared across these datasets. Since the lineage origin (approximately in the mid-20th century), a more persistent and stable viral population was estimated in Asia and China, while in Europe and Thailand, a sharp increase following the introduction (i.e., 2005 and 2007, respectively) of GI-19 was observed, succeeded by a rapid decline. Although a greater number of sites on the S1 subunit were under diversifying selection in the Asian and Chinese datasets, more focused and stronger pressures were evident in both the European (positions 2, 52, 54, 222, and 379 and Thai (i.e., positions 10, 12, 32, 56, 62, 64, 65, 78, 95, 96, 119, 128, 140, 182, 292, 304, 320, and 323) strains, likely reflecting a more intense and uniform application of vaccines in these regions. This evidence, along with the analysis of control strategies implemented in different areas, suggests a strong link between effective, systematic vaccine implementation and infection control. However, while the overall evolutionary rate was estimated at approximately 10^−3^ to 10^−4^, a significant inverse correlation was found between viral population size and the rate of viral evolution over time. Therefore, despite the stronger selective pressure imposed by vaccination, effectively constraining the former through adequate control strategies can efficiently prevent viral evolution and the emergence of vaccine-escaping variants.

## 1. Introduction

RNA viruses are characterized by a high evolutionary rate, enabling them to rapidly explore the evolutionary landscape [1]. Recombination is also commonly reported in RNA viruses, especially positive-sense ones, further expanding their evolutionary potential [2]. The consequences for biological flexibility, and consequently for human and animal health, are well documented: host jumps, the emergence of antiviral resistance, and the evasion of host immunity, to name just a few [3,4]. However, a high mutation rate merely provides a substrate for evolution; other forces are required to select variants with potentially higher fitness. Natural selection is a key driver in the evolution of organisms, including viruses. Its influence is consistent as long as the product of natural selection and effective population size is much greater than 1 (s∙Ne >> 1, where s is the selection coefficient and Ne is the effective population size). Thus, an interplay of various conditions occurs, largely influenced by the environment in which the organism exists.

In the world of viruses, the host is the primary and most significant determinant, affecting the strength of selective pressures and constraining the size of the viral population. Additionally, environmental factors impacting the host population, such as structure, contact network, and infection susceptibility, act at a higher hierarchical level, further influencing the outcome of viral evolution. In livestock production, managerial and control measures, which vary significantly between and within geographical areas, also contribute to this determinism.

Infectious bronchitis virus (IBV), a member of the species *Avian coronavirus*, order *Nidovirales*, suborder *Cornidovirineae*, family *Coronaviridae*, subfamily *Orthocoronavirinae*, genus *Gammacoronavirus*, and subgenus *Igacovirus* (https://ictv.global/taxonomy; accessed on 12 January 2024), is one of the most significant viral pathogens in poultry, responsible for substantial worldwide economic losses due to both direct and indirect costs. IBV primarily causes upper respiratory tract disease, which can lead to high mortality in the presence of secondary infections, and certain strains also cause nephritis. The genital tract of layer and breeder birds can also be affected, leading to reproductive disorders and altered egg production [5]. The viral genome, approximately 27 kb in length, encodes both non-structural (such as the RNA-dependent RNA polymerase (RdRp) and other accessory and regulatory proteins) and structural proteins (i.e., the spike, envelope, membrane, and nucleocapsid proteins) [6]. The spike protein (S), particularly the S1 subunit, is extensively studied due to its role in receptor attachment and cell tropism, and as the primary target of the host immune response, including neutralizing antibodies and cell-mediated immunity [6,7,8,9,10]. Therefore, the S1 region is a valuable target for investigating the evolutionary forces acting on the viral genome and phenotype. Moreover, because of its high genetic heterogeneity due to both high mutation and recombination rates [11,12,13], the S1 region is the most commonly sequenced one for both classification purposes and molecular epidemiology analysis. Currently, the most recognized classification scheme involves the differentiation of IBV strains into genotypes, further divided into lineages, based on genetic distance thresholds and phylogenetic analyses of the S1 region [9].

Specifically, the GI-19 lineage of IBV was selected for the present study for several reasons. Following its origin in Asia, it spread throughout the ‘Old World’, particularly in Europe, where it successfully and persistently established itself in diverse environments and production systems, becoming the dominant field lineage [13]. Due to its significant economic impact, it has been subjected to intense monitoring, resulting in a considerable number of well-annotated sequences. Consequently, various control strategies have been implemented to mitigate its impact, although the chosen approaches have varied considerably both between different regions and within them [14,15]. The effects of different intervention strategies, especially vaccination, on the population dynamics and evolution of the GI-19 genotype, have been independently assessed at a local scale [14,16], demonstrating the biological significance of the managerial systems on the evolution of GI-19 IBV. However, there is a lack of data on the interaction of these dynamics with other forces on a broader scale. Finally, with IBV being a coronavirus, insights into its evolutionary patterns and driving forces may provide valuable information for a better understanding and control of other human and animal coronaviruses.

## 2. Materials and Methods

### 2.1. Dataset

Complete S1 sequences, for which the collection country and year were available, were downloaded from GenBank. To identify those belonging to the GI-19 lineage, all sequences were iteratively aligned with the GI-19 reference sequences provided by Valastro et al. [9]. Sequences exhibiting a genetic distance greater than 13% from all reference strains were excluded. Clustering within the GI-19 lineage was further confirmed by aligning the selected sequences with the reference dataset provided by Valastro et al., using Translator X [17]. To account for the coding nature of the S1, the alignment was performed at the amino acid level using the MAFFT method [18], and then the nucleotide sequence was superimposed. A phylogenetic tree was reconstructed based on this alignment using IQ-TREE [19], with branch support assessed by performing 1000 bootstrap replicates. The best substitution model was selected based on the Bayesian Information Criterion (BIC), calculated using the same software. The occurrence of recombination events among selected sequences was evaluated using GARD [20], implemented in Datamonkey [21].

The preliminary phylogenetic analysis revealed consistent geographic clustering, predominantly with European and Asian strains clustering on separate branches, supported by high bootstrap values. Based on this evidence, two separate datasets were generated, including exclusively Asian and European sequences, respectively. Exceptions, such as rare Asian strains clustering within the European clade, were removed from the dataset, assuming they represented recent reintroductions that have not evolved in the specific area for an extended period.

**Figure 1:**
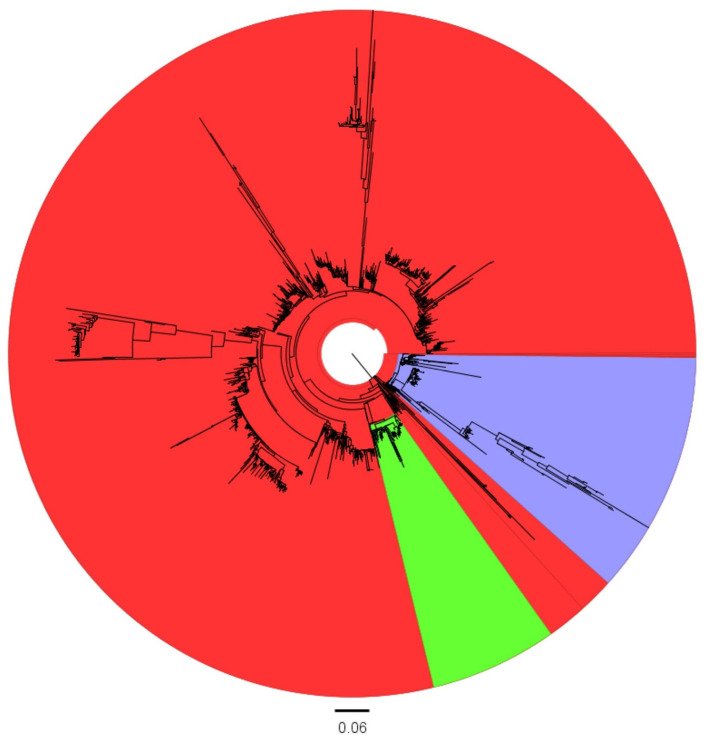
Maximum likelihood phylogenetic tree based on the selected S1 sequences. The Asian, European, and Thai clades are highlighted in red, blue, and green, respectively.

Furthermore, to assess whether the virus’s propensity to evolve is attributable to cluster-specific features rather than environmental conditions, sub-clades of the Asian group were also considered, and dedicated datasets were created. Specifically, datasets exclusively comprising Chinese and Thai sequences were generated. These countries were selected because they provided an adequate number of sequences forming a monophyletic clade on the phylogenetic tree, indicative of independent evolution (Figure 1).

### 2.2. Population Dynamics and Evolution Reconstruction

The time to the most recent common ancestor (tMRCA), evolutionary rate, and viral population dynamics were reconstructed using the Bayesian serial coalescent approach implemented in BEAST 1.10.4 [22]. The nucleotide substitution model was selected based on a BIC score calculated using JmodelTest2 [23]. The lognormal relaxed molecular clock method was chosen based on the marginal likelihood estimation through path-sampling and stepping-stone methods, as suggested by Baele et al. [24]. A non-parametric Bayesian Skygrid [22] was implemented to reconstruct the viral population dynamics (i.e., relative genetic diversity: effective population size × generation time; Ne × τ) over time. Since the Asian strains were largely over-represented, the original dataset was downsized for comparability with the European dataset. To this end, six independent datasets were generated by random sampling without replacement, selecting up to four Asian sequences per year. This approach also allowed for the evaluation of the sampling effect on result robustness [25]. Similarly, for the China–Thailand comparison, the Chinese dataset was subsampled, limiting the most recent sequences to 2017, when the last Thai sequence was available.

The analyses were performed independently on each of the defined datasets by conducting a 100-million-generation Markov Chain Monte Carlo (MCMC) run, and sampling population parameters and phylogenetic trees every 10 thousand generations. Results were accepted if the Estimated Sample Size (ESS) was higher than 200, and the mixing and convergence, visually inspected using Tracer 1.7 [26], were adequate. The results were summarized in terms of mean and the 95% highest posterior density (95HPD). Maximum clade credibility (MCC) trees were constructed and annotated using TreeAnnotator (BEAST package). Additional summary statistics and graphical outputs were generated using custom R scripts with the R libraries ggplot2, treeio, ggtree, and ips [27,28].

For each dataset, the average evolutionary rate for each branch of the MCC tree was correlated with the viral population size over time using rolling window correlations between the two regular time series. The statistical significance of the rolling correlation coefficients was estimated, accounting for multiple testing effects via Monte Carlo simulations by permuting one of the variables while keeping the other fixed, as implemented in the NonParRolCor [29] library of R. Briefly; the test is based on Monte Carlo simulations, permuting the dependent variable under analysis, and keeping the independent variable fixed. A critical value for the rolling correlation coefficients was calculated for each window length, enabling a comparison with the correlation coefficients of each window length. The Pearson correlation coefficient was calculated, setting the statistical significance to *p* < 0.05 (i.e., the 95th quantile of the critical value). Different window lengths were evaluated, ranging from 3 years in length to the entire study duration, in 2-year increments, and the correlation coefficient and significance were assessed for each window.

### 2.3. Selective Pressure Analyses

The action of selective pressures for each dataset (i.e., Europe vs. Asia, and China vs. Thailand) was estimated using methods based on calculating the difference between non-synonymous and synonymous substitution rates (dN/dS). The presence and intensity of pervasive and episodic diversifying selection were assessed using FUBAR [30] and MEME [31], respectively. A site-by-site comparison of the forces acting on each amino acid position of the S1 protein was performed using the contrast-FEL method [32]. This approach identifies individual alignment sites where two (or more) sets of branches in a phylogenetic tree have different dN/dS ratios. In this study, the evolutionary patterns of Asia vs. Europe and China vs. Thailand were compared. Thus, branches of the phylogenetic tree leading to European or Thai strains were selected as the foreground. All analyses were performed using HyPhy [33]. The required phylogenetic trees were reconstructed using IQ-TREE based on the same codon alignment used for selective pressure analysis.

The statistical significance level was set to *p* < 0.05 for MEME and a posterior probability >0.9 for FUBAR. The false discovery rate (FDR)-corrected q-value of 0.1 was considered for contrast-FEL.

### 2.4. Homology Modeling

To evaluate the location and distribution of sites under selective pressure, the nucleotide sequence of a GI-19 strain S1 subunit was translated at the amino acid level, and the SWISS-MODEL web server [34] was used to identify the best template with an experimentally determined quaternary structure. The same program was used to estimate the protein structure through a homology-modeling approach. The obtained model was visualized and edited with Chimera [35], plotting the evolutionary features of the protein.

## 3. Results

### 3.1. Dataset

A total of 1802 GI-19 sequences, originating from 28 countries over the period 1993–2022, were included in the final dataset. Of these, 1612 originated from Asian countries and 181 from Europe. The list of sequences included in the study, along with the available metadata, is provided in the Supplementary dataset. No statistically significant evidence of recombination was detected among the strains included in the study.

The phylogenetic analysis demonstrated distinct geographical clustering of Asian and European sequences, with few exceptions. Additionally, within the Asian cluster, which was primarily composed of Chinese strains, a monophyletic Thai cluster was present (Figure 1 and Supplementary tree), allowing the definition of four independent datasets for further analysis.

### 3.2. Reconstruction of Viral Population Dynamics and Evolution

**Figure 2:**
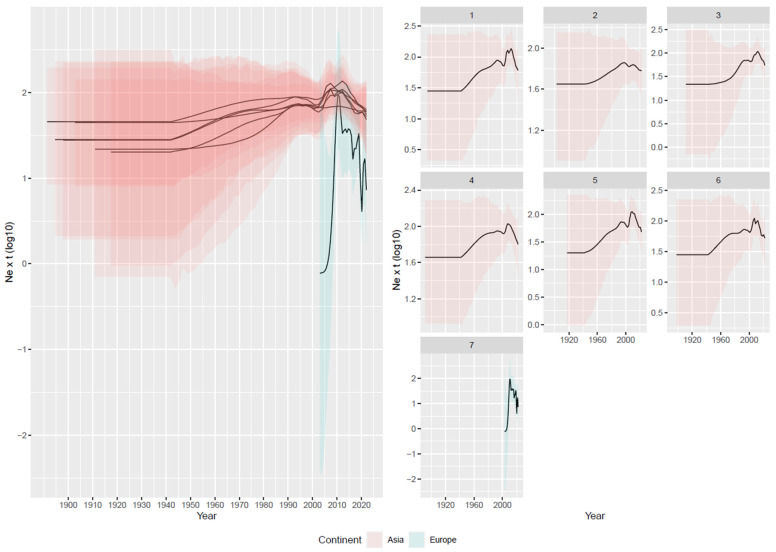
Left figure: mean and upper and lower 95HPD values of relative genetic diversity (Ne × t) of the Asian (red) and European (blue) GI-19 populations are reported over time. Right figure: mean and upper and lower 95HPD values are reported for each run.

The coalescent-based reconstruction of the population history of Asian GI-19 strains indicated a relatively ancient origin, approximately in the mid-20th century (tMRCA averaged across runs: mean = 1940.85 [95HPD = 1899.8–1979.7]), albeit with minor variations among randomly generated datasets (Supplementary Figure S1). Inference on population dynamics also led to consistent results among datasets (Figure 2). A progressive increase in relative genetic diversity was observed from the tMRCA until around 2010, with a temporary decline occurring around 2000. Thereafter, a decreasing trend continued until the end of the study.

**Figure 3:**
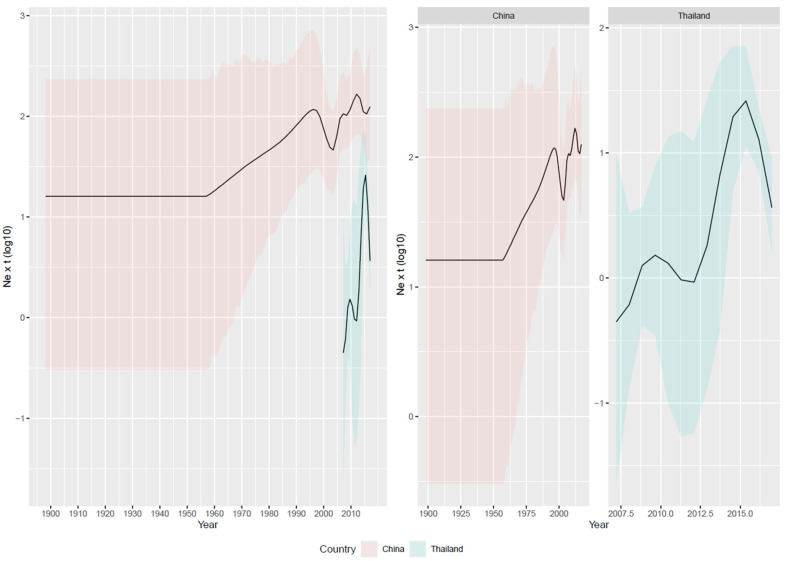
Depiction of relative genetic diversity over time of the GI-19 lineage was calculated based on the Chinese and Thailand datasets. Mean values are represented as a black line, while 95HPD intervals have been displayed as red (China) or blue (Thailand) shaded areas.

The European dataset revealed a more recent introduction (about 2005) followed by an abrupt expansion and subsequently a quick and marked decrease (Figure 2 and Supplementary Figure S1). When analyzing only the Chinese sequences, the estimated tMRCA and population dynamics largely mirrored the overall Asian trend, characterized by a progressive increase from the tMRCA (mean = 1948.64 [95HPD = 1897.12–1980.88]) until 2000, followed by a brief decline and then a major peak in 2010, after which the viral population began to decrease (Figure 3). The introduction of the virus into Thailand was tentatively estimated around 2007 [95HPD: 2006.56–2007.94]; the viral population size progressively increased, with minor fluctuations until 2015, and then significantly declined thereafter (Figure 3).

**Figure 4:**
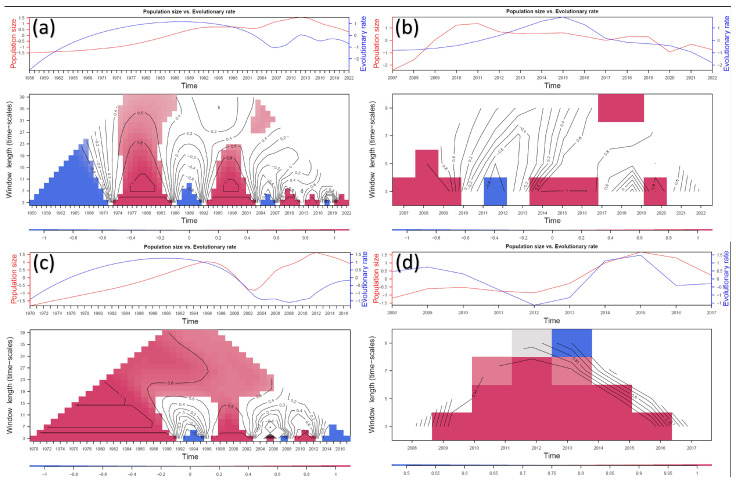
Results of the rolling correlation coefficient analysis among viral population size and evolutionary rate for the (**a**) Asian, (**b**) European, (**c**) Chinese, and (**d**) Thai datasets. For each dataset, the upper panel reports the trend (centered and scaled) of both variables while in the bottom one, the heatmap reports the rolling correlation coefficients calculated for different years and window sizes. Coefficients that are not statistically significant (95% confidence level) are blank. The strength of the correlation has been color-coded. Line contours indicate similar values of rolling correlation coefficients. To increase the resulting robustness, analyses were performed starting from the mean estimation of tMRCA.

The overall evolutionary rate was estimated at approximately 10^−3^ to 10^−4^, regardless of the considered dataset (Supplementary Figure S1). However, when examining the branch-specific substitution rate, a certain time dependency was observed (Supplementary Figures S2 and S3). A rolling window correlation analysis between viral population size and evolutionary rate confirmed a positive and statistically significant correlation, with an increasing evolutionary rate corresponding to higher population sizes and vice versa. However, this association, although generally consistent, did not always reach statistical significance for the entire considered period and was even reversed (i.e., negative correlation) in certain time windows. When testing windows of different lengths, a stronger correlation was observed for shorter time intervals across all observed datasets (Figure 4).

### 3.3. Selective Pressures Analysis

**Figure 5:**
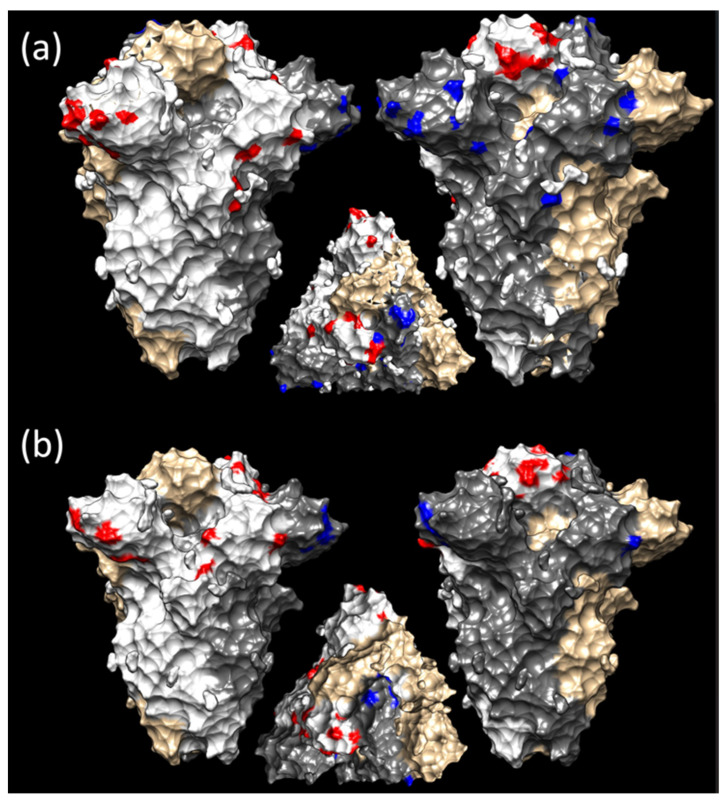
Different views of the quaternary structure of the IBV spike protein. The S1 regions have been edited to highlight different selective pressure features. (**a**) The sites under episodic diversifying selection in the Asian (red) and European (blue) datasets have been reported in the white and grey-colored monomer. The ochre monomer has been reported to depict the overall spike structure. (**b**) The same color scheme has been used to compare China (red) and Thailand (blue). A more detailed representation of the overall protein structure is reported in Supplementary animations S1 and S3. Images have been generated with the SWISS-MODEL web server and edited with Chimera.

The FUBAR analysis detected 25 sites under pervasive diversifying selection in the Asian dataset and 13 in the European one (Supplementary data). However, 41 sites were identified under episodic diversifying selection among Asian sequences, and 32 among European sequences, using MEME (Figure 5a, Supplementary data, and Supplementary animation S1). A limited number of codons were found to be under the same pressure in different datasets; specifically, FUBAR detected codons 58, 64, 65, 132, 331, and 389, and MEME codons 58, 62, 65, 89, 96, 120, 315, 321, 333, 389, 415, 483, and 522 (Supplementary data).

**Figure 6:**
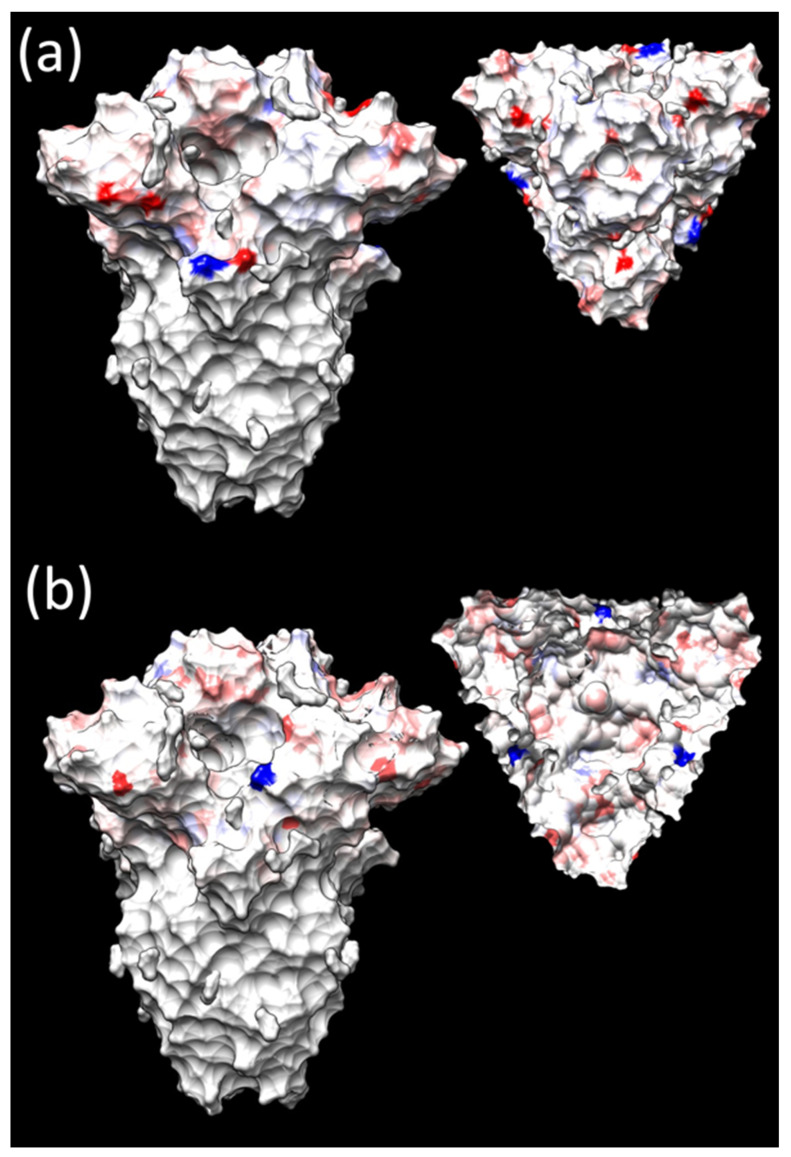
Different views of the quaternary structure of the IBV spike protein. The S1 regions have been edited to highlight different selective pressure features. The dN/dS difference between (**a**) Europe and Asia and (**b**) Thailand and China datasets, calculated using FUBAR, is reported using a continuous color scale ranging from positive (red) to negative (blue) values. A more detailed representation of the overall protein structure is reported in Supplementary animations S2 and S4. Images have been generated with the SWISS-MODEL web server and edited with Chimera.

Despite the higher number of sites under diversifying selection in the Asian dataset, when comparing dN/dS site by site between the two datasets, the European one exhibited overall stronger diversifying selective strength, especially on the S1 surface (Figure 6a and Supplementary animation S4). This was confirmed via contrast-FEL analysis, which detected different actions of diversifying selection at seven sites. In five sites (i.e., 2, 52, 54, 222, and 379), a higher non-synonymous substitution rate was estimated among European lineages, and in two sites (i.e., 22 and 167) among Asian ones. Sites exposed on the S1 surface were involved in this case as well.

The evaluation of the Chinese and Thai datasets revealed 12 and 31 S1 positions under pervasive diversifying selection by FUBAR (Supplementary data), respectively, while 34 and 20 were estimated under episodic diversifying selection by MEME (Supplementary data, Figure 5b, and Supplementary animation S3). Sites 39, 58, 65, 119, 120, 167, 282, 390, 437, and 484 were detected by FUBAR in both datasets, while 65, 120, 167, 288, 292, 321, 322, 383, 390, and 484 were detected by MEME (Supplementary data). An overall stronger selective pressure was detected acting on the viral surface among Thai strains (Figure 6b). Accordingly, all 18 sites (i.e., 10, 12, 32, 56, 62, 64, 65, 78, 95, 96, 119, 128, 140, 182, 292, 304, 320, and 323) detected under differential diversifying selection by contrast-FEL were under stronger pressure in the Thai dataset.

## 4. Discussion

Historically, the epidemiology of IBV has been marked by the emergence of new genetic variants with varying levels of virulence and epidemiological characteristics. While certain variants have remained confined to specific geographic regions or faded quickly, others have demonstrated the capacity to spread across extensive distances and maintain their presence over prolonged periods. [9,36,37]. The GI-19 lineage, believed to have originated in China decades ago, had subsequently spread to other Asian countries, Europe, and Africa [13]. The present study not only confirms the ancient origin of GI-19 but it also suggests an even earlier emergence than previously believed. Interestingly, despite its long history and capacity to migrate long distances, we observed a strong geographical clustering that separates Asian from European strains (the areas where most sequences were obtained). In contrast to the remarkable local dispersal, rare long-distance or intercontinental introduction events were inferred. This distribution pattern may be explained by the requirement for relatively direct contact, unlikely to occur between countries lacking strong commercial or cultural ties [38]. However, a small Asian clade stemming from the European one was present (Supplementary Figure S1). While not directly relevant to the purpose of this study, this finding may reflect a previously observed pattern for IBV lineage GI-16 (Q1) [39], and its epidemiological implications and potential developments should be considered and monitored.

The presence of such distinct clustering and the occurrence of a monophyletic European clade (indicative of a single introduction event) enabled the investigation of the evolutionary patterns and dynamics of strains circulating independently in different environments. A similar scenario was also present within Asia, with a broad Thai clade originating from the Chinese one. This double comparison allowed us to evaluate whether comparable dynamics acted locally and to assess whether the propensity of viruses to evolve is attributable to cluster-specific features rather than environmental or managerial conditions. The Asian and European clades showed a substantially comparable evolutionary rate, and despite a slightly higher mean value for the European strain (Figure 2), its biological relevance seems unlikely. Therefore, the evolutionary potential of the two groups can be considered similar. However, the viral population dynamics of the two clades over time differed significantly. Across all randomly generated datasets, the Asian clade was characterized by a progressive increase in population size (albeit with a minor fluctuation around 2000), peaking around 2010, and moderately decreasing thereafter. This pattern can be attributed to a slow but steady expansion in the affected host population, indicative of limited interconnection among Asian locations (i.e., leading to slow viral dispersal) combined with poorly effective control measures (i.e., leading to a progressive increase in viral population size). Supporting this hypothesis, the decline observed around 2000 might be explained by enhanced monitoring activities and the implementation of stricter biosecurity measures against avian influenza, along with related control strategies [40,41]. The subsequent rapid rebound in the GI-19 population size might be attributable to the intensification of the Asian poultry industry, leading to higher animal densities, movements, etc., occurring in the same period. This intensification may have created conducive conditions for the virus’s spread and multiplication, underscoring the complex interplay between agricultural practices and viral epidemiology. In contrast, the history of the European GI-19 population was much more turbulent. A sharp increase occurred following the viral introduction, enhanced by the strong interconnection of the poultry production system and the absence of commercial barriers within the common European market. Thereafter, a comparably rapid decline was estimated, likely due to the implementation of effective control measures. In Europe, vaccination against IBV is routine and, despite an astonishing variety of protocols being applied in different countries, production companies, or even farms [15], in most instances, a heterologous vaccination with vaccines based on genotype GI-1 (Mass) in combination with another genotype (commonly GI-13, also known as 793B) is used. Such strategies have proven effective in controlling GI-19 infection both in experimental and field conditions [42,43]. Additionally, since approximately 2015, effective homologous GI-19-based vaccines have become available and started to be consistently applied in the field [14]. The prompt response of the European farming system, motivated by the impact GI-19 lineage had on poultry production, led to a reduction in the outbreak occurrence and viral circulation as well [14].

Although IBV vaccination is now commonly applied in Asian countries, some substantial differences occur. Initially, GI-19 emergence and circulation occurred when such efficient vaccination strategies were not yet developed or could not be extensively applied in Asia. GI-19’s persistence and slow expansion may have been facilitated by the existence of a less developed, rural sector characterized by inadequate biosecurity, managerial, and economic resources. A more accurate evaluation of the situation in China could provide better insights into the relationship between control strategies and viral epidemiology. The first GI-13 (strain 4/91) vaccine was introduced into this market around 2000. Initially, the combination 793B plus Mass was applied only to breeders, while a low percentage of broiler and layer farms adopted this approach, aiming to limit high costs. In 2009, a more affordable, non-proprietary GI-13-based vaccine was introduced and progressively gained market share in those categories as well.

It is noteworthy that a minor decline in the GI-19 population was observed around 2000, while a persistent decline was observable in the Asian population (largely represented by Chinese strains) after 2010. Moreover, in 2012, the first QX vaccine, namely the LDT-3 A strain, was introduced. However, it was only at the end of the decade, with the introduction of IB QXL-87 in 2018, which eventually gained the most market share, that the homologous protection strategy became predominant. Finally, a new GI-13-based vaccine (based on 1/96 strain) was licensed in 2019, further boosting the vaccination campaign. A sharp decline in the Asian viral population was observed at the same time. Therefore, it is highly probable that only the systematic application of either homologous or heterologous vaccination, which has been extensively proven to successfully control GI-19 infection and clinical signs [14,44], was effective. Single non-homologous vaccination or partial population coverage, even with combined heterologous vaccination, were ineffective or only slowed down viral circulation. Interestingly, the viral decline was much slower compared to the European estimations. In China, several companies shifted to locally produced, less expensive vaccines and decreased vaccination application intensity and standardization to reduce vaccination costs, during the warm season when the disease burden is perceived as lower. Such a strategy was proven ruinous in Italy under field conditions [14]. Despite the above-mentioned scenario not being representative of the entire Chinese farming system, the persistence of such pockets of suboptimal protection likely explains the prolonged viral persistence and dispersal. Further support emerges from the comparison between China and Thailand. In Thailand, Mass plus 793B-based vaccines are extensively applied [45]. Accordingly, the population dynamics in this Asian country overlap with the European ones, featuring an abrupt increase after viral introduction followed by a comparably sharp decline, when the GI-19 population in China was still stable or increasing. Notably, homologous GI-19 vaccination was first applied in Thailand after 2017, thus confirming that combined, heterologous vaccination, if widely and properly applied, can effectively control GI-19 challenges in field conditions. The different immune environments also affected the strength of selective forces. Despite a higher number of sites under diversifying pervasive or episodic selection being detected in Asian sequences, when the selective pressures were compared in a site-by-site fashion, more intense forces acted on the S1 subunit of European strains. While this might seem counterintuitive, a higher number of sites under significant but lower strength might be due to a larger viral population persisting for a long time under more diverse environmental conditions, necessitating adaptation to multiple, variable scenarios. In addition to different vaccination strategies, numerous IBV variants have been reported to circulate in China, contributing to a heterogeneous immune environment. Conversely, IBV genetic variants, farming conditions, including management, poultry breeds, vaccination approaches, etc., are more homogeneous in Europe, leading to more focused, stronger pressures. For example, the extensive vaccine administration, especially of homologous ones, in a high-turnover short-life animal population might pose more intense but less diverse evolutionary pressures compared to the highly heterogeneous natural immunity [16]. The fixation of non-synonymous mutations providing a fitness advantage through immune-escape mechanisms can be enhanced. Accordingly, most of the sites under differential selective pressure between the two datasets were located on the viral surfaces and in epitopic regions or sites involved in receptor binding [7]. Of note, most of the sites under diversifying pressures were located in the S1 hypervariable regions [46,47,48,49], stressing the role of immune pressure as a driver of IBV spike protein variability. The analysis of the Thai clade supports this hypothesis. Stronger selective pressures were estimated in this case as well. The intensively applied vaccination, combined with the predictably lower variability of the host and viral populations located in a smaller territory, justifies more homogeneous forces, resulting in stronger pressures on specific sites.

These findings raise the question of whether vaccination has side effects that enhance viral evolution. The presence of strong selective pressures (i.e., selective coefficients) is essential for natural selection to act. However, the role of population size (i.e., effective population size) cannot be neglected, as natural selection acts mainly on large populations. When correlating the evolutionary rate with the viral population size over time using a rolling window approach, a predominantly significant positive correlation was consistently detected, regardless of the dataset and time period considered. Effectively reducing the viral population size significantly curtails the evolutionary potential of IBV. Therefore, a properly planned vaccination strategy should be encouraged not only for its immediate clinical benefits but also to hinder viral evolution. Conversely, ineffective vaccination, resulting in lower selective coefficients while allowing ongoing viral circulation and evolution in a ‘vaccinated environment,’ should be seen as a major long-term threat. This scenario may lead to the emergence of immune-escape variants, a phenomenon widely acknowledged in the context of antimicrobial resistance [50,51,52]. A higher correlation was generally observed when narrower time windows were considered. This suggests a strict association and rapid response to changes in population dynamics. Although this demonstrates the flexibility and dynamism of IBV (and other coronaviruses) evolution, it also presents opportunities, particularly the rapid effectiveness and benefits of optimizing control strategies, both from clinical-economic and epidemiological-evolutionary perspectives.

Unfortunately, this study is not without limitations. Primarily, we acknowledge that within each considered macro-area, the variability in production systems can be significant, and further sub-regionalization might have provided different insights. However, the required high-resolution information was not available for either the environmental variables or the sampled strains. Nevertheless, we aimed to investigate the evolutionary pathways on a broad scale, considering that local ones were already evaluated [14,16]. Additionally, even if detailed information on the context of strain collection was available, it could still be misleading since a strain identification in a specific location does not imply that it evolved in that environment for an extended period. The introduction of a virus that had been evolving in a completely different scenario is possible, potentially biasing the estimations. A randomly sampled strain can reasonably be expected to evolve, on average and most of the time, under the selective pressures prevalent in the majority of farms in the region. Therefore, in the absence of longitudinal monitoring of the individual strain evolution over time and space, we consider our approximation adequate and representative of the phenomenon under investigation.

As a final note, the presence of such strong geographical clustering, with monophyletic clades persisting in environments characterized by differential selective pressures, should be carefully monitored. Similar to what happens in higher organisms, where the founder effect, isolation, and adaptation to new environments may lead to speciation events, such compartmentalization could be an intermediate step in the emergence of new IBV lineages. Since currently recognized lineages are separated by long branches, indicative of ancient divergence, the real-time emergence of new lineages has yet to be observed and remains one of the primary unanswered questions in the study of IBV evolution.

## 5. Conclusions

The present study addresses the complex interplay of determinants that contribute to shaping the evolution of GI-19, and likely, other IBV strains and coronaviruses in general. The structure and management of farming systems, along with the applied control strategies and their variation over time, have significantly affected the population dynamics of GI-19 and exerted differential selective forces. In areas where more intense, homogeneous, and organized control strategies could be implemented, a prompt and remarkable decrease in GI-19 circulation was achieved, despite the predictable increase in selective pressures. Moreover, these strategies effectively constrained the virus’s evolutionary potential. The identification of monophyletic clades, which are geographically determined and evolving under different selective pressures, deserves further monitoring and investigation since this could represent a crucial step in the emergence of new IBV lineages. Understanding this process may aid the comprehension of the long-term evolution of this class of viruses and represent a prerequisite for preventing it.

## Data Availability

All sequence accession numbers and relative metadata are available in the Supplementary data file.

## Supplementary Materials

The following supporting information can be downloaded at https://www.mdpi.com/article/10.3390/v16030481/s1: Supplementary Figure S1. Boxplots of the tMRCA (upper figure) and evolutionary rate (lower figure) posterior probability. Results of the randomly generated Asian dataset and the European one are reported. Supplementary Figure S2. Plot depicting the estimated evolution rate for each branch (color-coded) of the MCC tree (left figure). Additionally, a scatterplot reporting the branch location over time and the relative evolutionary rate has been reported (right figure). A smoothed conditional means and relative 95 confidence intervals have also been superimposed. The results of the independent Asian datasets are reported on pages 1 to 6, with the European results on page 7. Supplementary Figure S3. Plot depicting the estimated evolution rate over time for the randomly generated Asian datasets and the European ones. The independent runs have been color-coded while the 95HPD intervals are depicted as a shaded area. Supplementary data. List of sequences included in the present study and relative metadata. Raw results of different selection pressure estimation analyses are reported for different datasets and methods. Supplementary tree. Maximum likelihood phylogenetic tree. All metadata, strain labels, and statistics (e.g., bootstrap support) are reported.
