# Supplementary material for: The Effect of Global Spread, Epidemiology, and Control Strategies on the Evolution of the GI-19 Lineage of Infectious Bronchitis Virus
Source: Viruses. 2024 Mar 20;16(3):481. doi: 10.3390/v16030481 (PMC10974917; doi:10.3390/v16030481)
Supplement: Supplementary file 1 [file viruses-16-00481-s001.zip › Supplementary Figure 2.pdf]

1

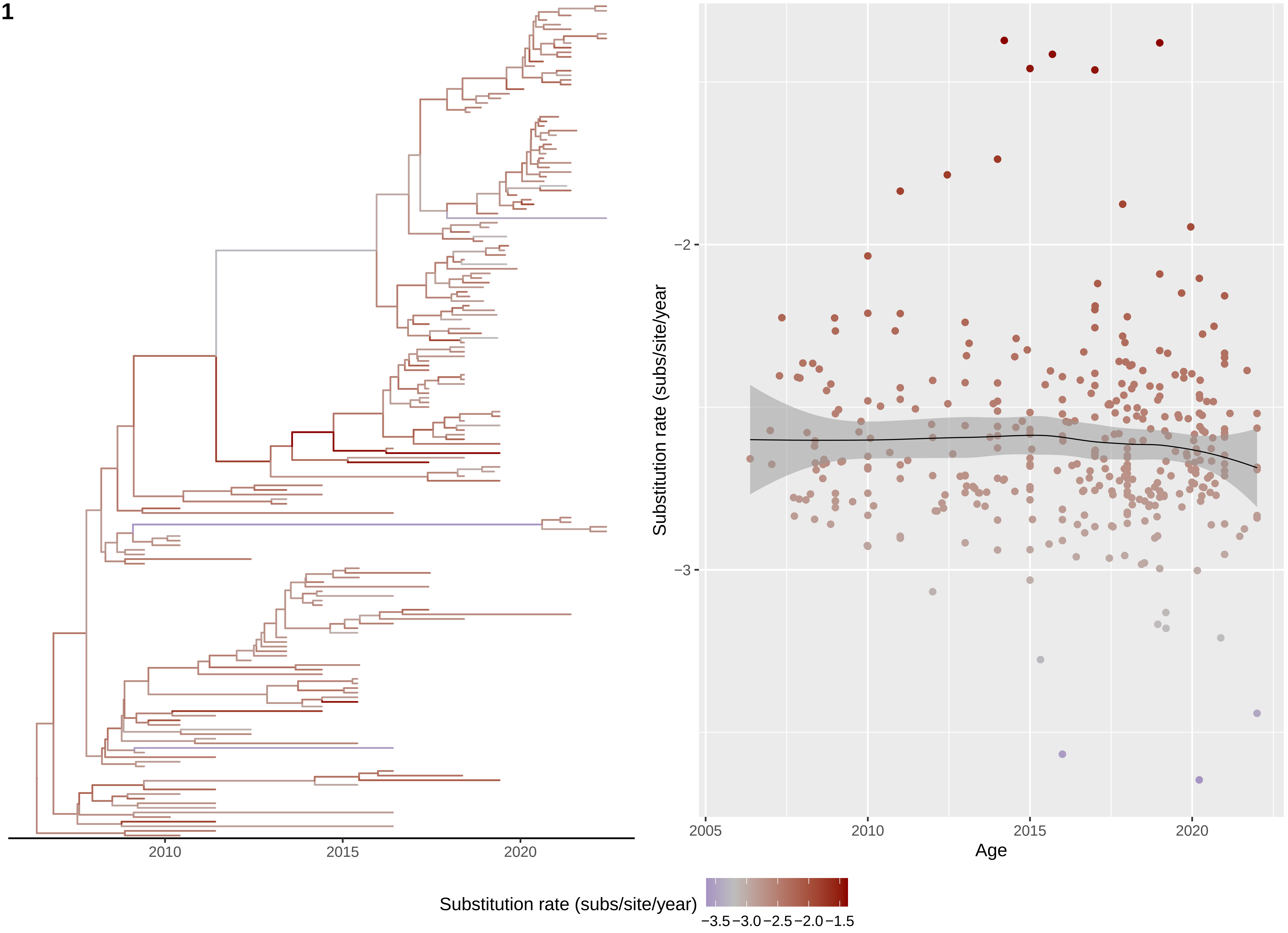

2010
2015
2020
Substitution rate (subs/site/year
−2
−3
2005
2010
2015
2020
Age
Substitution rate (subs/site/year)
−3.5 −3.0 −2.5 −2.0 −1.5

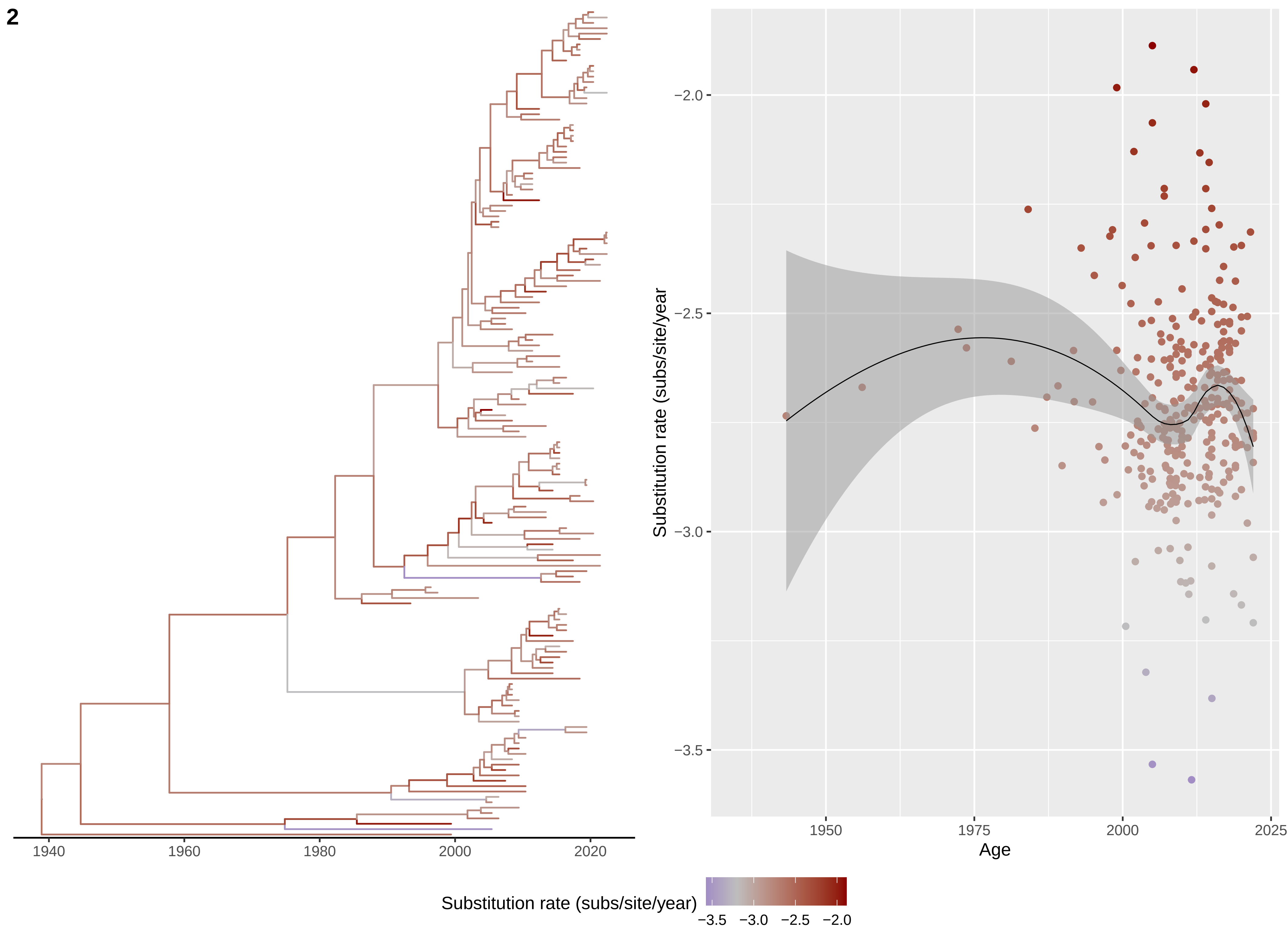
2
1940
1960
1980
2000
2020
−2.0
−2.5
−3.0
−3.5
Substitution rate (subs/site/year)
1950
1975
2000
2025
Age
Substitution rate (subs/site/year)
−3.5
−3.0
−2.5
−2.0

3

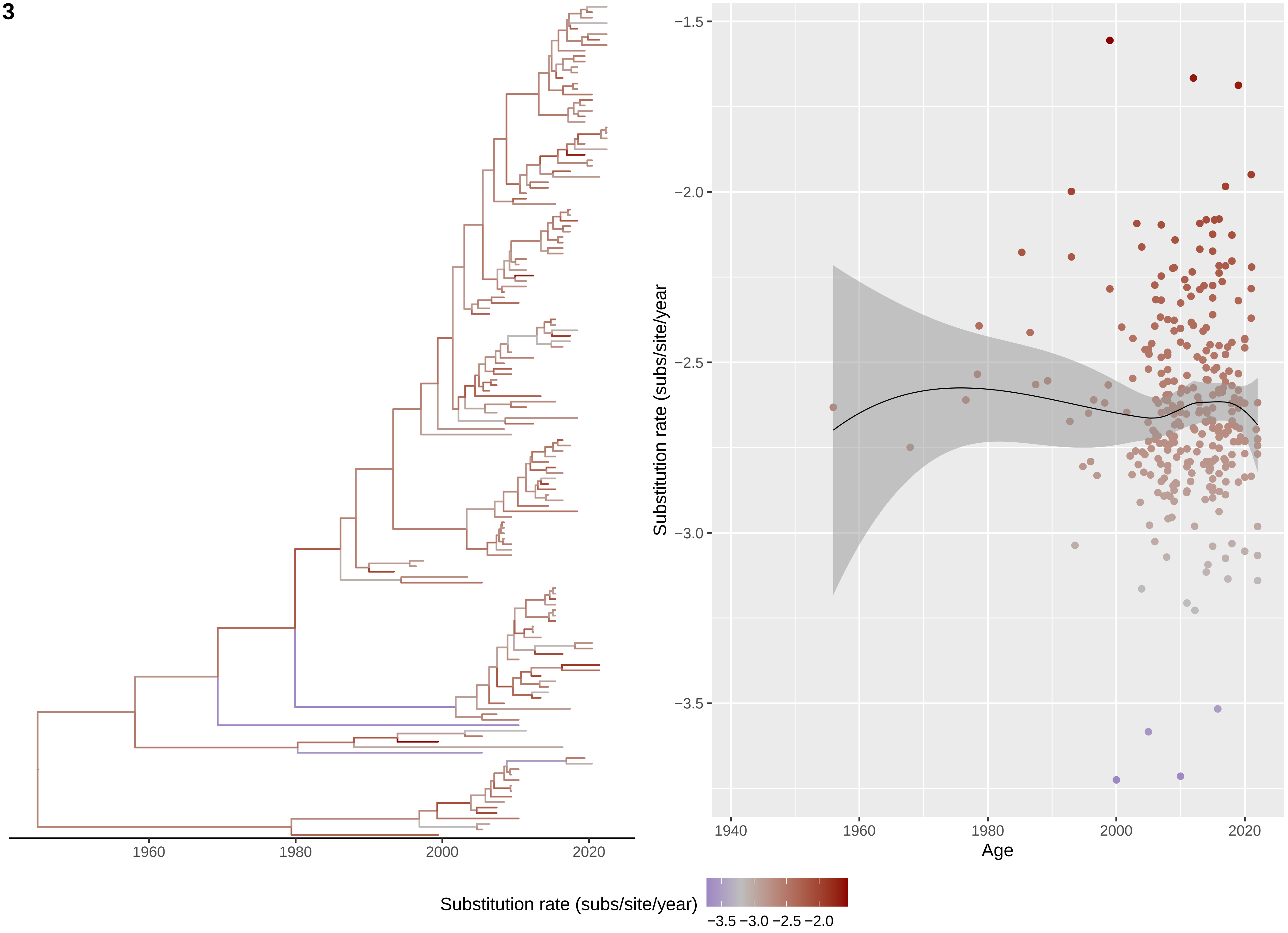
1960
1980
2000
2020
−1.5
−2.0
−2.5
−3.0
−3.5
Substitution rate (subs/site/year)
1940
1960
1980
2000
2020
Age
Substitution rate (subs/site/year)
−3.5 −3.0 −2.5 −2.0

4

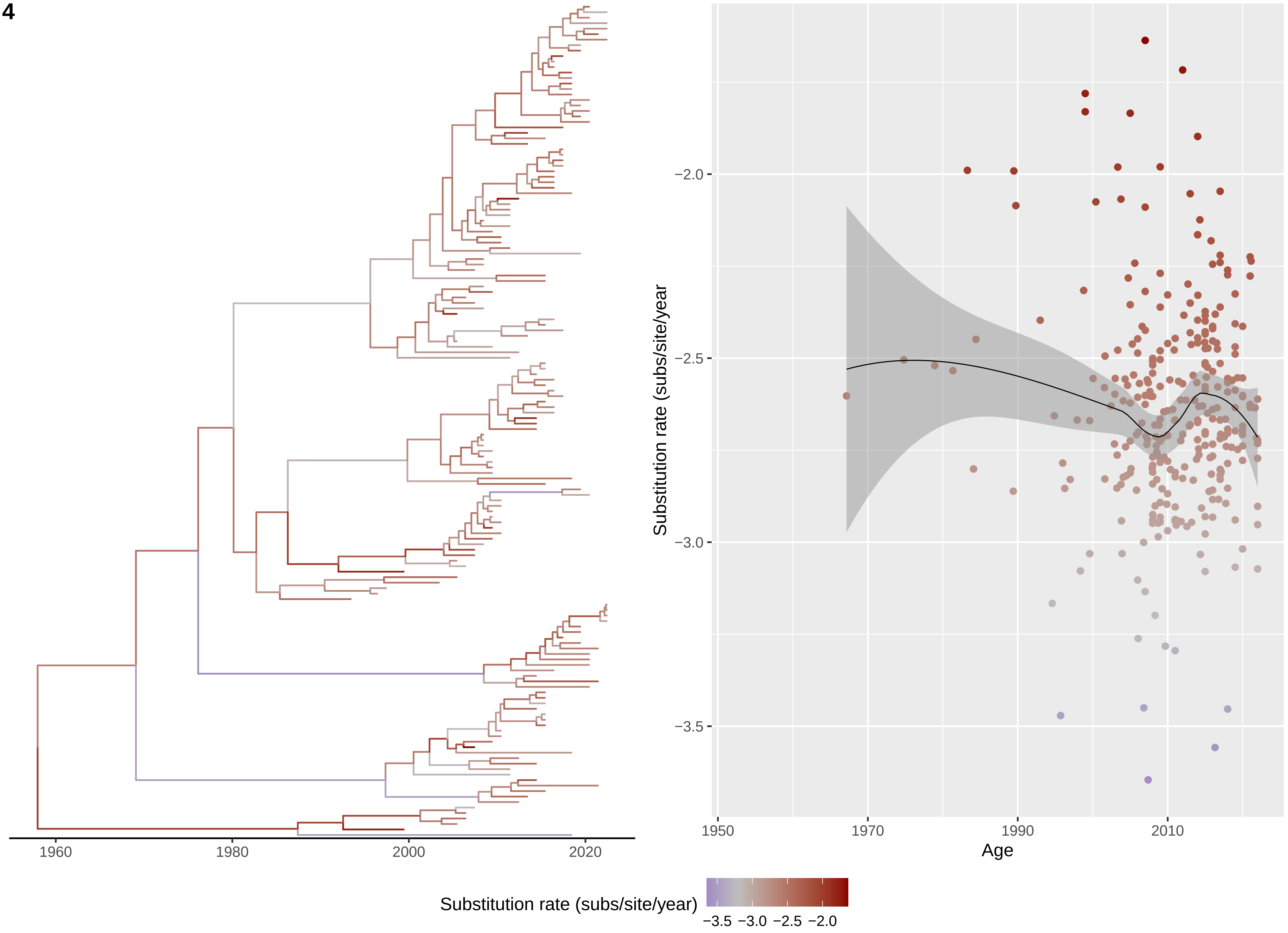
1960
1980
2000
2020
−2.0
−2.5
−3.0
−3.5
Substitution rate (subs/site/year
1950
1970
1990
2010
Age
Substitution rate (subs/site/year)
−3.5 −3.0 −2.5 −2.0

5

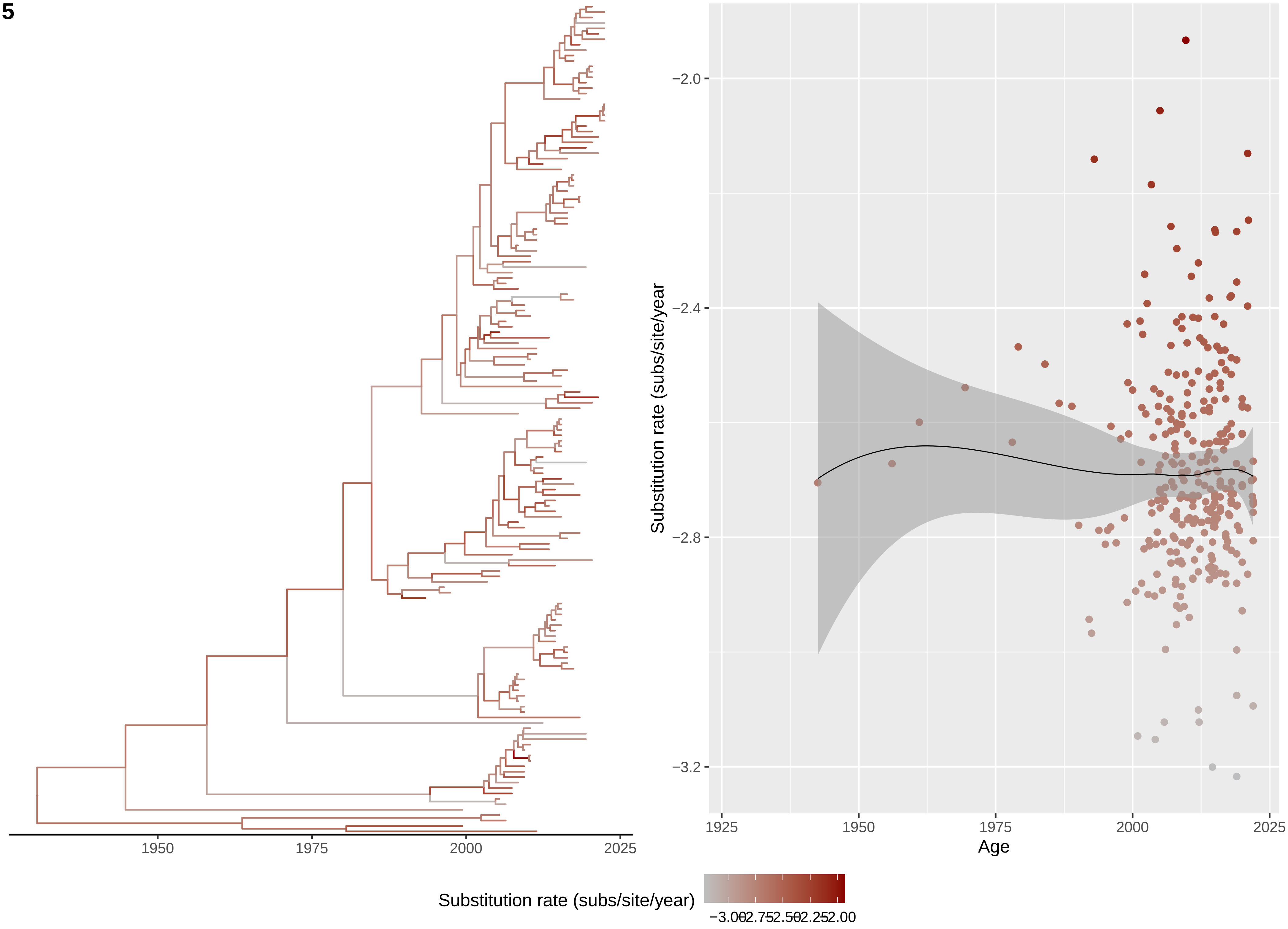
1950
1975
2000
2025
Substitution rate (subs/site/year)
−2.0
−2.4
−2.8
−3.2
1925
1950
1975
2000
2025
Age
Substitution rate (subs/site/year)
−3.00
−2.75
−2.50
−2.25
−2.00

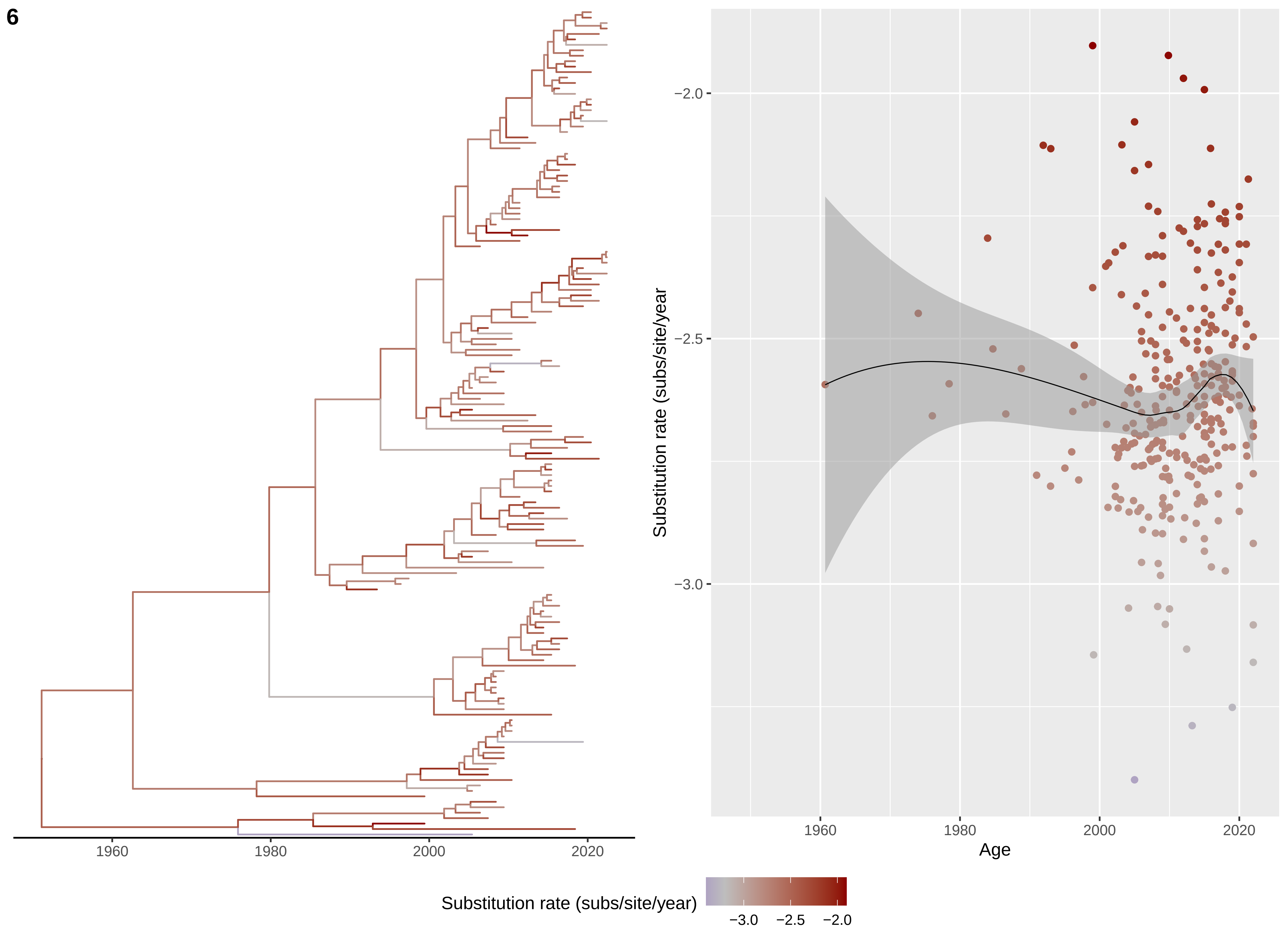
6
1960
1980
2000
2020
−2.0
−2.5
−3.0
Substitution rate (subs/site/year)
Age
Substitution rate (subs/site/year)
−3.0
−2.5
−2.0

7

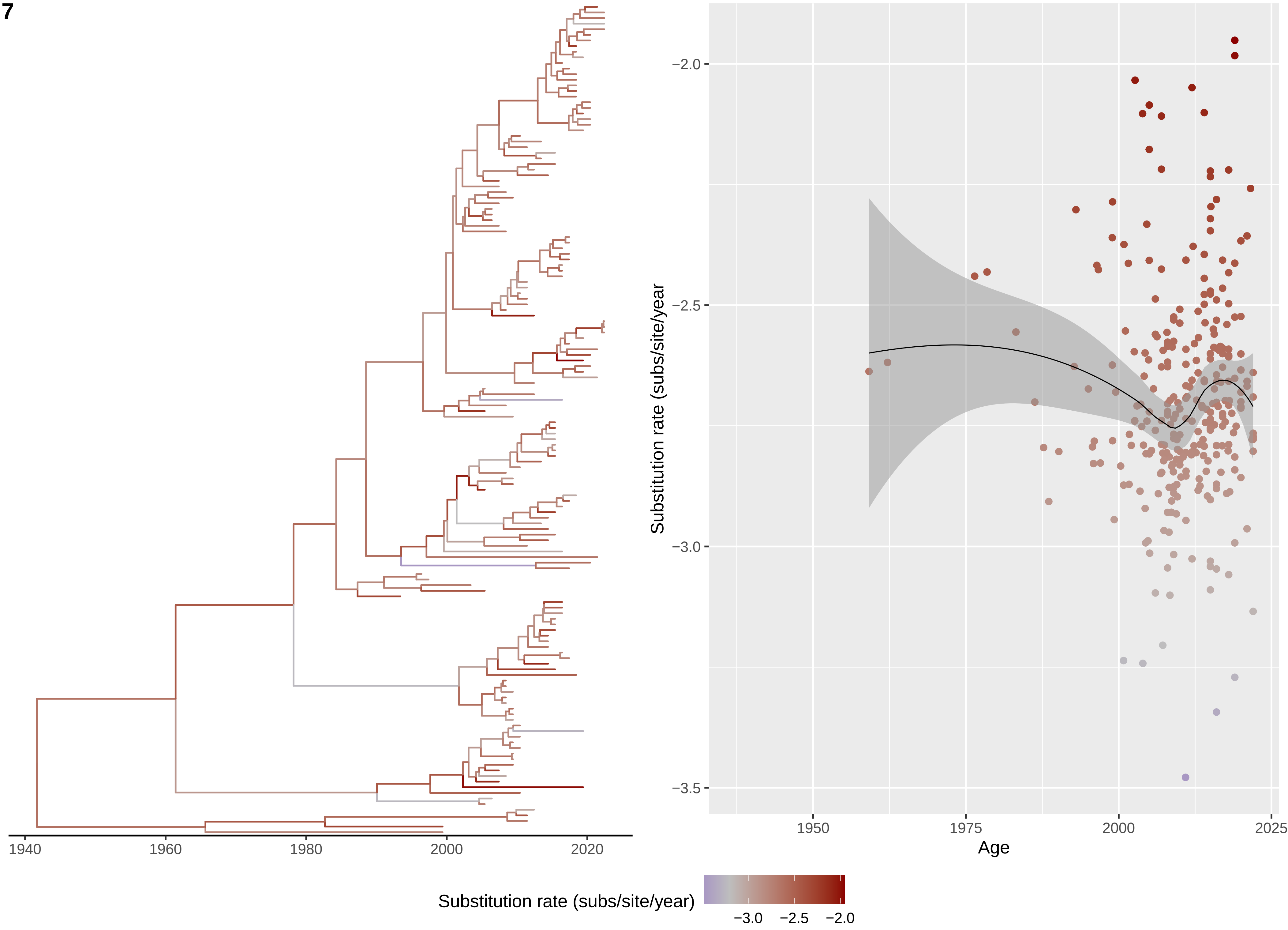
1940
1960
1980
2000
2020
−2.0
−2.5
−3.0
−3.5
Substitution rate (subs/site/year
1950
1975
2000
2025
Age
Substitution rate (subs/site/year)
−3.0
−2.5
−2.0
