# Supplementary figures and images for: The Effect of Global Spread, Epidemiology, and Control Strategies on the Evolution of the GI-19 Lineage of Infectious Bronchitis Virus
Source: Viruses. 2024 Mar 20;16(3):481. doi: 10.3390/v16030481 (PMC10974917; doi:10.3390/v16030481)

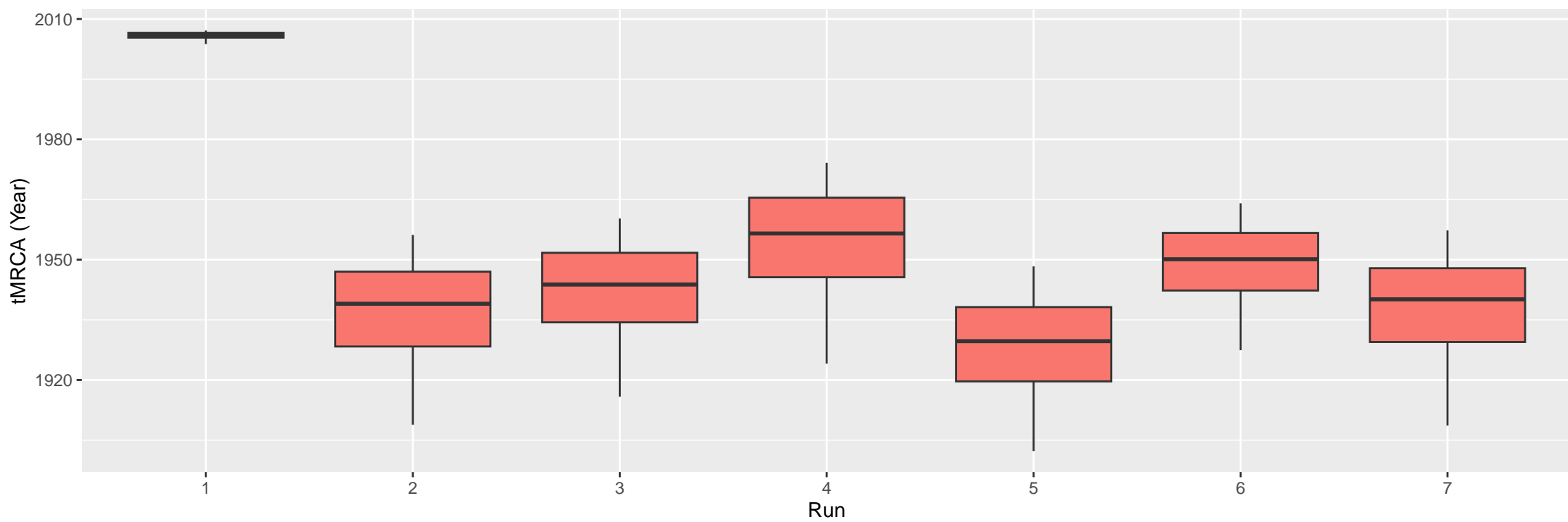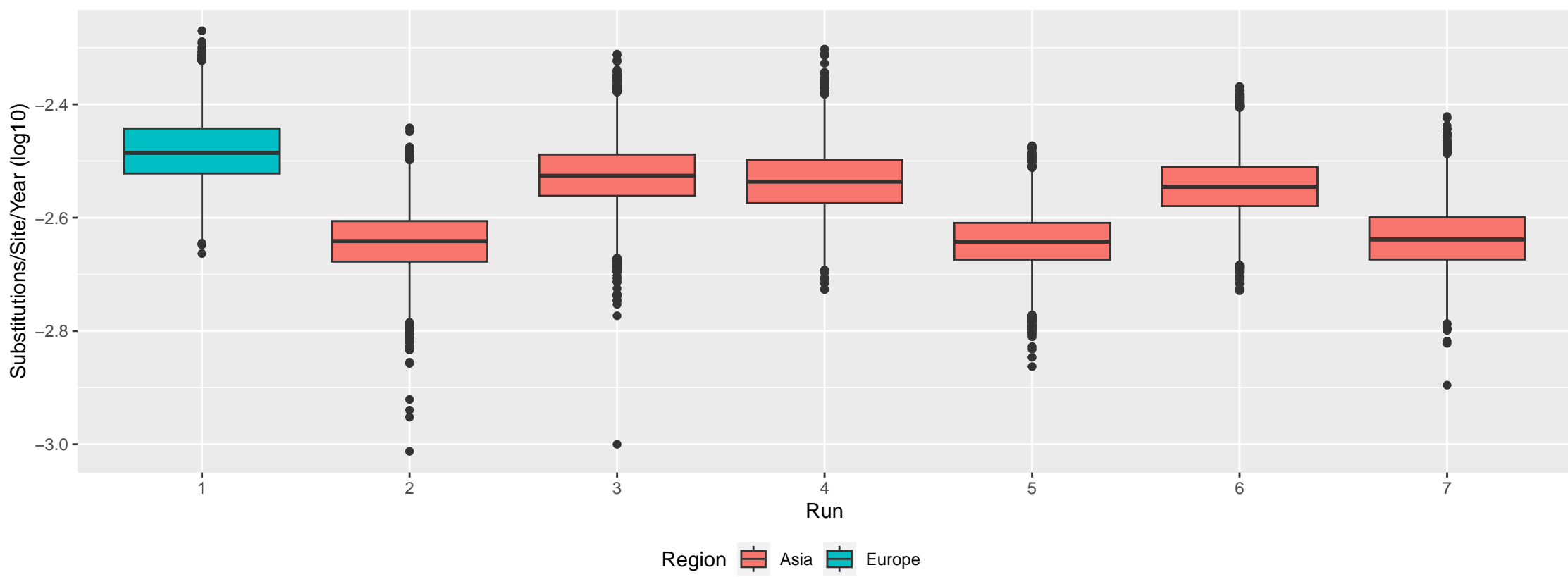

Supplement: Supplementary file 1 [file viruses-16-00481-s001.zip › Supplementary Figure 1.pdf]

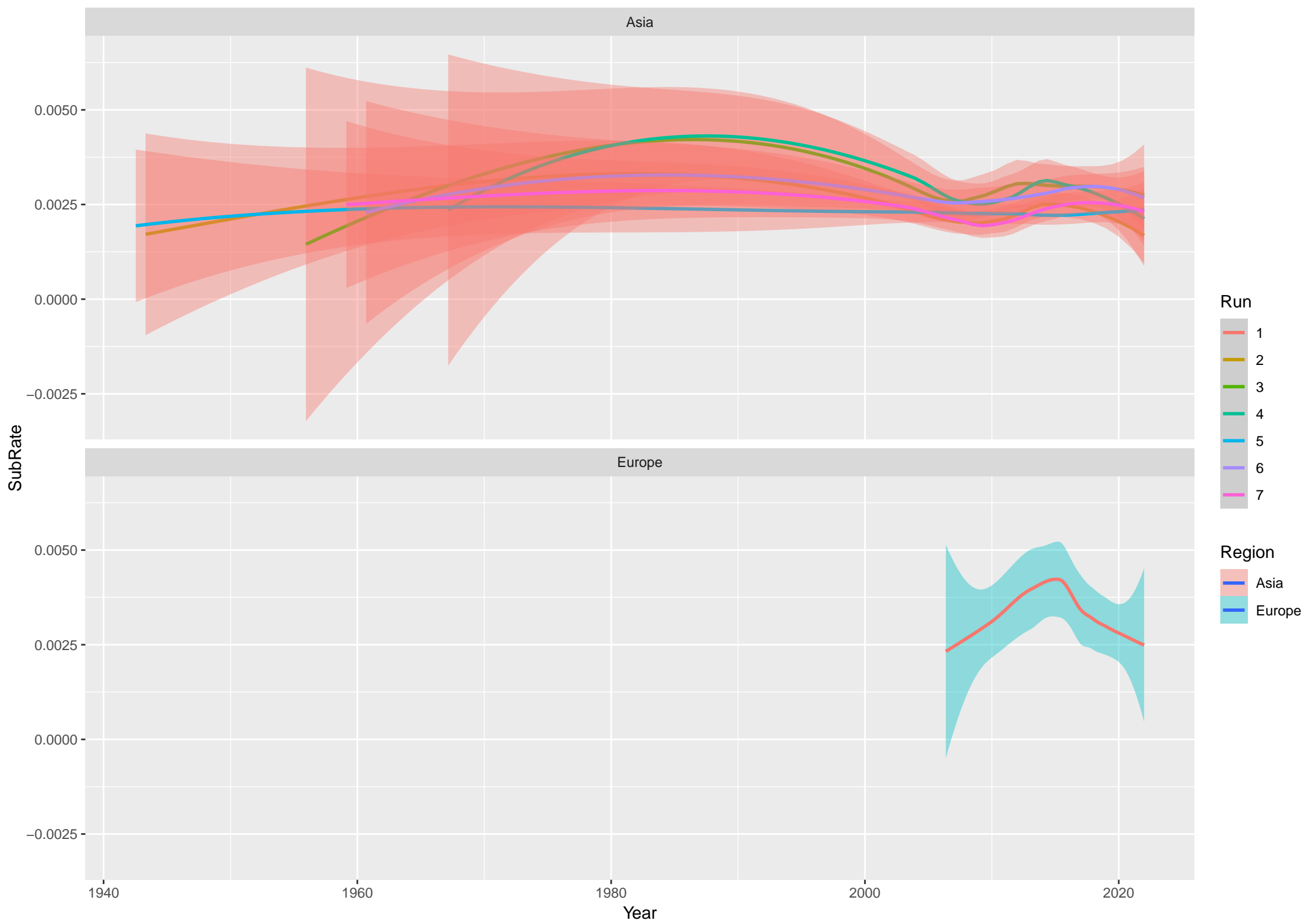

Supplement: Supplementary file 1 [file viruses-16-00481-s001.zip › Supplementary Figure 3.pdf]
